# Longitudinal microbiome profiling reveals impermanence of probiotic bacteria in domestic pigeons

**DOI:** 10.1371/journal.pone.0217804

**Published:** 2019-06-17

**Authors:** Kirsten Grond, Julie M. Perreau, Wesley T. Loo, A. James Spring, Colleen M. Cavanaugh, Sarah M. Hird

**Affiliations:** 1 Department of Molecular and Cell Biology, University of Connecticut, Storrs, Connecticut, United States of America; 2 Department of Organismic and Evolutionary Biology, Harvard University, Cambridge, Massachusetts, United States of America; 3 Department of Integrative Biology, University of Texas at Austin, Austin, Texas, United States of America; 4 Independent Researcher: Sutton, Massachusetts, United States of America; University of Illinois, UNITED STATES

## Abstract

Probiotics are bacterial species or assemblages that are applied to animals and plants with the intention of altering the microbiome in a beneficial way. Probiotics have been linked to positive health effects such as faster disease recovery times in humans and increased weight gain in poultry. Pigeon fanciers often feed their show pigeons probiotics with the intention of increasing flight performance. The objective of our study was to determine the effect of two different probiotics, alone and in combination, on the fecal microbiome of Birmingham Roller pigeons. We sequenced fecal samples from 20 pigeons divided into three probiotic treatments, including prior to, during, and after treatment. Pre-treatment and control group samples were dominated by Actinobacteria, Firmicutes, Proteobacteria, and Cyanobacteria. Administration of a probiotic pellet containing *Enterococcus faecium* and *Lactobacillus acidophilus* resulted in increase in average relative abundance of *Lactobacillus* spp. from 4.7 ± 2.0% to 93.0 ± 5.3%. No significant effects of *Enterococcus* spp. were detected. Probiotic-induced shifts in the microbiome composition were temporary and disappeared within 2 days of probiotic cessation. Administration of a probiotic powder in drinking water that contained *Enterococcus faecium* and three *Lactobacillus* species had minimal effect on the microbiome. We conclude that supplementing Birmingham roller pigeons with the probiotic pellets, but not the probiotic powder, temporarily changed the microbiome composition. A next step is to experimentally test the effect of these changes in microbiome composition on host health and physical performance.

## Introduction

Vertebrates house large and diverse communities of commensal and pathogenic bacteria on and within their bodies, the “microbiome” (reviewed in [1,2]). Generally, the majority of these bacteria reside within the lower intestinal tract in numbers that can equal the total number of host body cells [3]. Many studies in model systems have found an important role for the gut microbiome in host health [4,5], and research has aimed to define the features of a healthy microbiome [4,6,7]. Imbalances in the microbiome, referred to as dysbiosis, are associated with a variety of human diseases including obesity and inflammatory bowel disease [8,9]. Several causes of dysbiosis have been described in mammals, including host genetic factors, pathogen infections, repeated antibiotic treatments, and changes in host diet [10–13]. The microbiome has been relatively well described in humans and mammalian model organisms, but remains poorly described in birds [14–16].

Probiotics are dietary supplements containing live microorganisms that are intended to replace or supplement a host’s current microbiome. They can potentially confer health benefits [17] and are one potential therapy for combating dysbiosis. The use of probiotics has surged in popularity over the past decade. Probiotics are commonly used in humans and poultry to combat gut dysbiosis associated with antibiotic treatment [18,19] and are also used as mental and physical performance enhancing supplements [20,21]. In mice, supplemental *Lactobacillus* species leads to the production of more radiant fur and reduces stress-induced corticosterone and anxiety-related behavior [22,23]. In poultry, probiotic use is associated with weight gain [24,25], and probiotics have even been suggested as a potential method for treating dysbiosis in captive raised endangered species [26] and disease mitigation in wildlife [27]. However, probiotic supplementation does not always have an effect on the gut microbiome and host health [28,29], and information on their effectiveness is especially sparse for domesticated non-poultry birds [30].

Probiotics are commonly used in the domestic pigeon circuit and are widely available commercially (J. Spring, personal communication). Birmingham Roller pigeons (*Columba livia domestica*) were originally bred for their ability to perform backward somersaults during flight [31]. This flight display has become the main activity for competitive Birmingham Roller shows. It is a common belief in the pigeon world that probiotic administration increases the Rollers’ flight performance (J. Spring, personal communication) but the effectiveness of probiotics in domestic pigeons has not been experimentally verified. How do probiotics affect pigeon health, performance, fitness, or physiology? A first step toward answering this question is to determine how probiotics affect the composition of the gut microbiome.

The objective of our study was to examine the effects of probiotics on the microbiome of Birmingham Roller pigeons. We collected time-series fecal samples from pigeons subjected to four probiotic treatments and analyzed the microbiome using high-throughput sequencing of a hypervariable region of the 16S rRNA gene. If probiotics alter the microbiome of pigeons in a directed way, we expect to see increased similarity in microbiomes within probiotic treatment groups. Conversely, if probiotics have little, or a random, effect on the microbiome, we expect to see no differentiation in microbiomes across probiotic treatment groups. Furthermore, if probiotics do produce a directed change in microbiome composition, defining the timeline for these changes will inform how long probiotic treatments should be administered before we can confidently assess probiotic effects on pigeon health or performance. Finally, determining how quickly probiotic bacteria are no longer detected in feces will inform whether probiotics should be administered continuously in order to have an effect.

## Methods

### Sampling design

**Fig 1:**
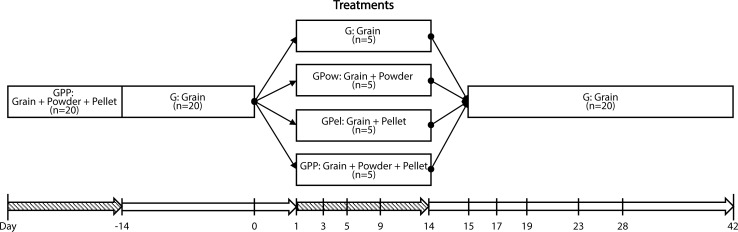
Sampling design showing probiotic treatments received by pigeons over a 42-day timeline. Timeline showing when pigeons were fed only Grain (white) and when birds were exposed to probiotic or control treatments (dashed). Prior to the experiments, birds were fed a Grain + probiotics diet, and the 14 days prior to experimental treatments all birds were fed a Grain-only, or control diet. Fecal samples collected between day 0 and 42, indicated by numbers below the treatments, were sequenced.

Domesticated Birmingham Roller pigeons were sampled on site at a pigeon fancier’s facility in Sutton, MA from October to November, 2015. Pigeons ranged from 3–5 months of age at the start of sample collection and had reached adult size. We tested the effect of two probiotics that are typically administered to domestic pigeons and were those used for the pigeons prior to our study: (1) probiotic pellets added to their food and/or (2) probiotic powder added to their water source (Table 1). Prior to our experiments, pigeons were kept in two aviaries and fed a diet of grain, probiotic pellets, and probiotic powder for 3 months (Fig 1). Because we used pigeons from a third party owner, we were unable to sample pigeons that had not been previously exposed to probiotics. Twenty pigeons were divided equally into four dietary treatment groups: a control group with no probiotics administered to either food or water (grain-only, G), grain with probiotic pellet added (GPel), grain with probiotic powder added to water (GPow), and grain with probiotic pellets and probiotic powder added to water (GPP; Table 1).

**Table 1:** Dietary composition of pigeon food and probiotics according to the manufacturer.

Dietary Component (Abbreviation)	Ingredients
Grain **(G)** (F.M. Brown’s Sons, Inc. Premium Pigeon Feed Breeder Kafir)	Popcorn, Milo, Winter Wheat, Canadian Peas, Maple Peas, Hulled Oats, Kafir
Probiotic Pellet **(GPel)** (Blue Seal Feeds, Inc. AVI PELS probiotic pellets)	*Enterococcus faecium*, *Lactobacillus acidophilus*, Yeast Fermentation Solubles
Probiotic Powder **(GPow)** **(**Probios Dispersible Powder, Multi-species)	*Enterococcus faecium*, *Lactobacillus acidophilus*, *Lactobacillus casei*, *Lactobacillus plantarum*
Grit	Granite Chips, Calcium Chips, Vitamins, Minerals

The grain-only treatment birds received 26 g of grain per bird per day and the probiotic pellet (Blue Seal Feeds, Inc. AVI PELS, Muscatine, IA, USA) treatment consisted of 12 g of probiotic pellets and 12 g of grain per bird per day. Pigeons consumed the full amount of food provided daily. For the probiotic powder (Probios, Chr. Hansen Inc, Milwaukee, WI) treatment, dispersible powder was used throughout the course of the experiment. 3.3g of probiotic powder was mixed into 1.25 liters of water (0.66g per bird) per day. All treatments received grit and water *ad libitum* as part of their diet. Since water was provided *ad libitum*, amount of water ingested was not measured. To minimize bias associated with genetic relatedness, we divided direct siblings over the four treatment groups. In addition, ages and sexes of birds were balanced across treatment groups. No probiotics were given to pigeons for two weeks prior to the experiment. Subsequently, pigeons were subjected to the treatment diets for two weeks and then fed no probiotics for another two weeks.

Pigeons were housed by treatment groups and separated into individual cages for collection of fecal samples. Pigeons were individually marked using metal leg bands enabling assignment of fecal samples to individuals. To prevent contamination, all materials used for feces collection were UV and bleach (10%) sterilized prior to use. Whole fecal samples were collected in cryovials and frozen in liquid nitrogen (-196°C) and stored at -80°C. Samples were collected on day 0 (prior to treatment), days 1, 3, 5, 9, and 14 (during treatment), and on days 15, 17, 19, 23, 28, and 42 (post treatment; Fig 1). In addition to fecal samples, a negative field control was also collected onsite.

### Extraction, PCR, and sequencing

Genomic DNA was extracted using the Purelink Microbiome Kit (Thermo Fisher Scientific—Invitrogen, Waltham, MA, USA) following manufacturer’s instructions. PCR reactions were performed in triplicate and pooled using NEB One*Taq* DNA Polymerase (New England Biolabs, Inc., Ipswich, MA, USA) and dual index primers [32](Integrated DNA Technologies, Coralville, IA, USA). PCR conditions consisted of 35 cycles of: 20 s at 94°C, 20 s at 55°C, and 15 s at 68°C preceded by an initial denaturing for 30 s at 94°C, and followed by a final extension for 5 min at 68°C. PCR products were purified using Agencourt AMPure XP (Beckman Coulter, Danvers, MA, USA) using a modified protocol [33]. DNA concentrations were quantified using the Qubit Assay (Life Technologies, Carlsbad, CA). The V4 regions of the 16S rRNA genes were sequenced using the Illumina MiSeq platform at the Harvard Medical School Biopolymers Facility.

### Sequence analysis

The DADA2 pipeline in R version 3.4.3 was used to process sequence data [34,35]. DADA2 calls operational taxonomic units (OTUs) from sequence-based microbial communities by performing stringent quality control steps and subsequently calling each unique amplicon sequence variant (ASV) an OTU. The program outputs an ASV table, which records the number of times each unique sequence variant is observed in each sample. This is in contrast to OTU calling by grouping sequences by percent sequence identify (e.g. 97%) and is a higher resolution method for bacterial OTU calling [34]. After quality assessment, sequences were trimmed to remove low quality read areas, paired-end sequences were merged and chimeras removed. Sequences were assigned taxonomically using RDP’s Naïve Bayesian Classifier [36] with the Silva reference database (v. 128) [37]. Sequences identified as chloroplast and mitochondria were removed from the dataset. A multiple alignment was generated using the *DECIPHER* package in R [38], and a phylogenetic tree constructed with the *phangorn* package version 2.4.0 [39]. Likely sequence contaminants were identified and removed using the *decontam* package in R [40], which identified contaminant ASV’s in the negative field control and PCR control. All further analyses were conducted using the cleaned sequence set.

### Statistical analyses

All statistical analyses were performed in R [35]. Two measures of alpha diversity, the observed number of ASV’s and the Shannon diversity index [41], were calculated using the *phyloseq* package [42]. Samples were rarefied to a depth of 1100 sequences prior to alpha diversity analysis, and samples with fewer sequences were removed. Analysis of Variance (ANOVA) was used to determine whether alpha diversity of probiotic treatments differed from the grain control treatment.

Microbiome community analysis was conducted using the *phyloseq* package and results were visualized using the ggplot2 package [42,43]. Non-metric Multidimensional Scaling (NMDS) analysis was applied to Bray Curtis, unweighted UniFrac and weighted UniFrac distances [44]. Community centroid location and cloud dispersion of weighted UniFrac distance matrices were compared among all fecal samples of all treatments and time points. To ensure homogeneity of sample variance prior to treatment, community centroid distance and community dispersion of all treatments were compared on day 0. In addition, treatments were compared on day 14, which represents the last day of treatment, and on day 28, which represents 14 days post treatment using the adonis function (PERMANOVA) from the vegan package [45].

Bacterial genera with known potential pathogenic or beneficial properties were identified in pigeon fecal samples to assess the effect of probiotics on specific members of the microbiome. Genera that were detected >100 times were identified and tested for effects of treatment on relative sequence abundance at day 0, 5, 15, and 23. We chose these sampling times to include prior to treatment (0), during treatment (5), immediately post-treatment (15), and 9 days post-treatment. Treatment relative abundances were compared to the grain control treatment using the TukeyHSD test, after establishing there was no significant variation in the grain control during the treatment period.

## Results

For the 20 pigeons studied, 232 fecal samples were sequenced, as well as a field negative control and PCR negative control. A total of 87 ASV’s were identified as contaminants by the *decontam* package in the negative field and PCR control and were removed from the data set (S1 Table). After this quality control, a total of 5,762,142 sequences remained for fecal samples (range: 30–117,236 seqs/sample). All samples with fewer than 1,000 reads were excluded, resulting in 207 fecal samples used in further analyses (for sample sizes per treatment/timepoint, see S2 Table).

### Community composition

**Fig 2:**
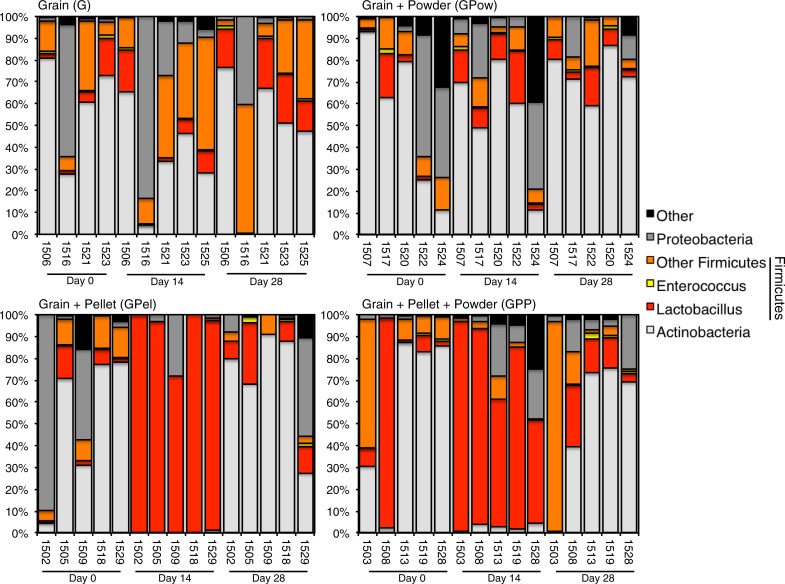
Relative bacterial phylum abundance in feces of pigeons before treatment (Day 0), on the last day of treatment (Day 14), and 14 days post-treatment (Day 28). Numbers on the x-axis represent pigeon ID. The Firmicutes phylum is depicted on a genus level.

The most common phyla detected in pigeons across treatments and time points were Actinobacteria (44.4%), Firmicutes (29.9%), Proteobacteria (21.0%), and Cyanobacteria (4.2%). We observed large individual variation between and within treatment groups (Fig 2 and S1 Fig). A shift towards a Firmicutes-dominated fecal microbiome was observed in both treatments that included the pellet probiotic: GPel and GPP. Prior to treatment (day 0) and 14 days post-treatment (day 28), fecal microbiomes of pigeons in the GPel treatment group were dominated by Actinobacteria first, followed by Proteobacteria and Firmicutes (Fig 2). At 14 days into treatment (day 14), all five individuals had fecal microbiomes dominated by Firmicutes, ranging from 71.8% to 99.9%. The increase in Firmicutes relative abundance almost exclusively consisted of an increase in *Lactobacillus* spp. (Fig 2). We detected similar shifts to a *Lactobacillus* spp. dominated community as detected in the GPel group in fecal microbiome composition of individuals assigned to the GPP treatment. We did not observe increased Firmicutes relative abundances in the Grain and GPow treatment on day 14 (Fig 2). Overall, we did not detect any effect of the GPow treatment on diversity and community composition of the pigeon gut microbiome. For this reason, further discussion focuses on treatments including the probiotic pellet (GPel and GPP).

**Fig 3:**
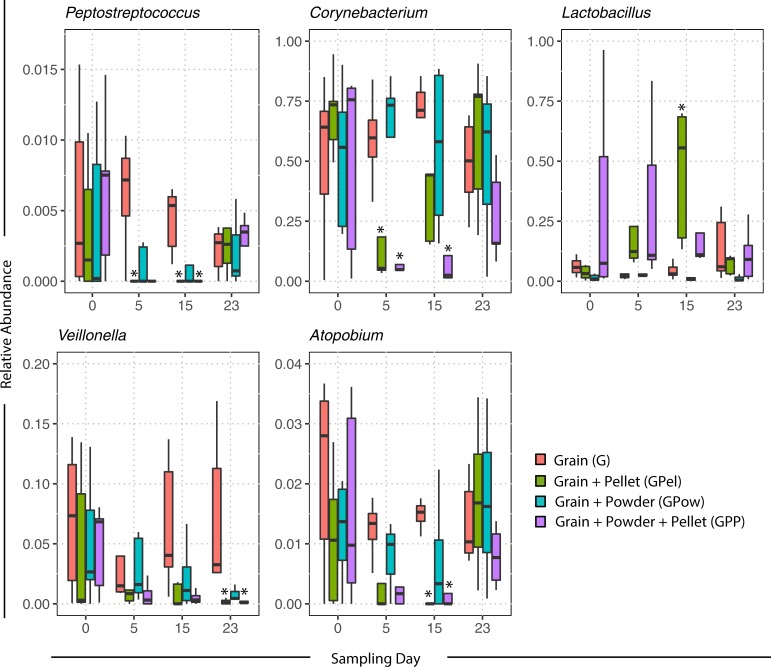
Relative abundance of bacterial genera before (day 0), during (day 5) and after treatment (day 15 & 23) with probiotic pellets and powder. Asterisks (*) indicate significant (α = 0.05) differences from the Grain (G) control treatment.

Relative abundances of five genera were significantly different from the control during the treatment period (Fig 3). Two of these genera include known human pathogenic species (*Peptostreptococcus* and *Corynebacterium*) [46,47], and two genera include species with known benefit to humans and birds (*Lactobacillus* and *Veillonella*) [46–49]. The function of the *Atopobium* genus is not known, but Atopodium spp. have been identified in the chicken gut microbiome [50]. *Peptostreptococcus* relative abundance was significantly lower than the Grain control in the GPP treatment at day 5 (p = 0.031), and in the GPel and GPP treatment on day 15 (p = 0.005; p = 0.007). *Corynebacterium* relative abundance was significantly lower than the control in the GPel and GPP treatments at day 5 (p = 0.013; p = 0.008), and in the GPP treatment at day 15 (p = 0.05). *Lactobacillus* relative abundance was significantly higher than the control in the GPel treatment at day 15 (p = 0.009), and *Veillonella* abundance was significantly lower in the GPel and GPP treatments at day 23. Last, *Atopobium* relative abundance in the GPel treatment was significantly decreased compared to the control (G; p = 0.027) on day 15, which was the day after treatment ceased.

### Diversity

#### Alpha diversity

**Fig 4:**
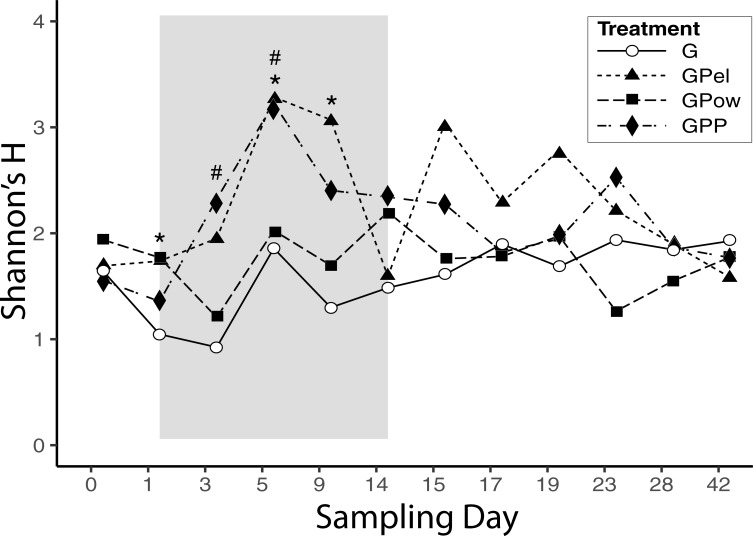
Bacterial diversity (Shannon Diversity Index) in fecal samples collected over 42 days from domesticated pigeons exposed to different diets. Shaded area represents treatment period, and treatments consisted of Grain (G), Grain + Probiotic Powder (GPow), Grain + Probiotic Pellet (GPel), and Grain + Probiotic Pellet + Probiotic Powder (GPP). Asterisks (*) represent a significant difference of the GPel treatment from the Grain (G) treatment, and pound signs (#) represent a significant difference of the GPP treatment from the Grain (G) treatment at α = 0.05.

Shannon’s diversity index and the number of ASV’s were consistently higher in the treatments that contained probiotic pellets (GPel and GPP) than in the grain-only control, but only rarely at a significance level of α = 0.05 (Fig 4 and S1 Fig, Table 2). Shannon’s diversity index was significantly higher than the Grain Control (G) in the Grain + Pellet (GPel) treatment on days 1, 5 and 9 and on days 3 and 5 in the Grain + Pellet + Powder (GPP) treatment (Table 2). The number of ASV’s in fecal samples from the Grain + Powder (GPow) treatment was significantly different from the Grain Control (G) on day 1, and the Grain Control differed significantly from the GPP treatment on day 5.

**Table 2:** Alpha diversity (Shannon’s H and number of Amplicon Sequence Variants (ASV’s) in fecal samples of Birmingham Roller pigeons exposed to different probiotic treatments. Diversity metrics that differed significantly from the control Grain treatment are bolded. Significance level was determined at α = 0.05.

	Grain (G)		Grain + Powder (GPow)				Grain + Pellet (GPel)				Grain + Powder + Pellet (GPP)			
Day	Shannon	ASV's	Shannon	p	ASV's	p	Shannon	p	ASV's	p	Shannon	p	ASV's	p
0	1.59	29.4	1.88	0.377	0.21	0.654	0.03	0.876	0.21	0.662	1.50	0.798	31.5	0.812
1	0.99	17.9	1.71	0.412	7.31	**0.043**	8.65	**0.032**	2.96	0.146	1.30	0.446	27.3	0.323
3	0.87	29.3	1.15	0.174	0.13	0.733	3.60	0.116	0.78	0.418	2.27	**0.020**	49.0	0.152
5	1.80	29.2	1.97	0.491	1.30	0.292	10.00	**0.013**	3.74	0.089	3.17	**0.001**	81.9	**0.001**
9	1.24	28.9	1.64	0.750	0.57	0.493	11.12	**0.016**	1.33	0.293	2.35	0.035	53.7	0.115
14	1.43	25.9	2.14	0.663	4.82	0.059	0.06	0.816	0.16	0.701	2.28	0.093	40.3	0.278
15	1.55	39.4	1.70	0.130	0.14	0.725	5.39	0.081	0.86	0.407	2.22	0.074	46.8	0.645
17	1.84	40.9	1.72	0.727	0.10	0.769	0.73	0.431	1.48	0.278	1.76	0.917	36.4	0.729
19	1.63	42.8	1.92	0.798	0.34	0.259	2.43	0.170	1.81	0.227	1.89	0.733	55.6	0.553
23	1.88	43.5	1.21	0.655	0.28	0.583	0.34	0.578	1.67	0.233	2.47	0.130	47.8	0.775
28	1.79	25.7	1.49	0.300	0.24	0.641	0.01	0.927	1.68	0.252	1.81	0.954	43.1	0.194
42	1.87	33.2	1.72	0.160	0.18	0.684	0.28	0.618	0.07	0.809	1.71	0.853	39.9	0.786

#### Beta diversity

**Fig 5:**
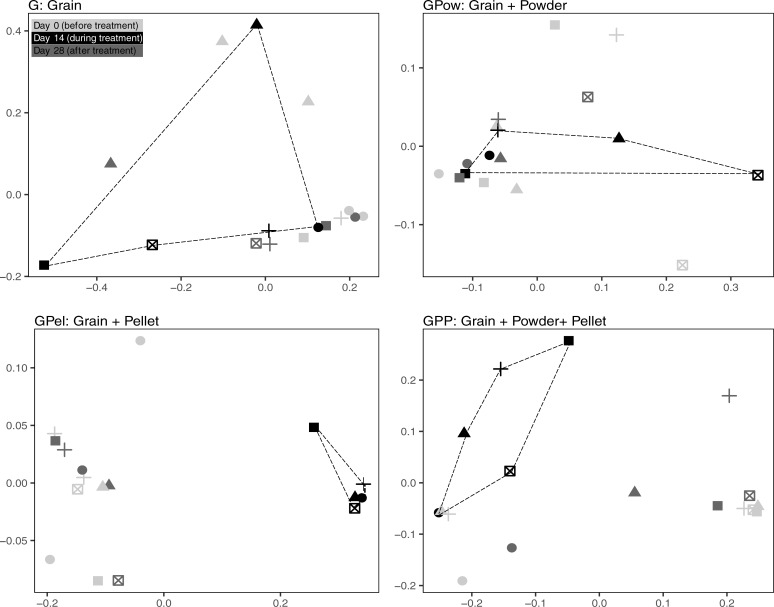
Non-multidimensional Scaling (NMDS) of weighted UniFrac distances from fecal microbiomes of domesticated Birmingham Roller pigeons. Samples collected before treatment (Day 0; Light grey), at the end of the treatment period (Day 14; Black), and two weeks post-treatment (Day 28; Dark grey). Symbols represent different individuals within the treatment. Lines connect the samples collected at day 14. Powder and Pellet refer to two different probiotic supplements the pigeons received during the treatment period.

We detected a shift in the gut microbial community after administration of probiotic pellets. On day 14, NMDS analyses of weighted UniFrac distances showed a clear visual differentiation within the fecal microbiomes of individuals that received the GPel and GPP treatments between day 14 and the other time points. This indicates that microbiomes of pigeons receiving treatments that included probiotic pellets (GPel, GPP) were distinctly different from microbiomes in the non-pellet treatments (GPow, G; Fig 5). Bray-Curtis distances showed similar patterns as weighted UniFrac (S3A Fig), but no clear separation of the GPel treatment was observed when using unweighted UniFrac distances (S3B Fig) Probiotic treatment was a significant driver of microbiome composition during treatment (day 14, PERMANOVA weighted UniFrac; F_3,16_ = 5.42, R^2^ = 0.504, p<0.001; see S3 Table for unweighted UniFrac and Bray-Curtis distances). No differentiation of fecal microbiomes was observed among treatments at day 0 and 28.

Overall, treatments differed significantly in centroid placement and cloud dispersion (F_3,228_ = 9.07, p < 0.001; F_3,228_ = 16.98, p < 0.001). Samples collected prior to treatment (day 0) and 14 days post-treatment (day 28) did not differ significantly among treatment groups with respect to centroid location and dispersion, indicating similar communities regardless of treatment (Day 0: F_3,23_ = 0.46–0.54, p = 0.807–0.698. Day 28: F_3,16_ = 0.95–0.82, p = 0.435–0.507). On day 14, the GPel treatment differed significantly from the Grain treatment in dispersion (TukeyHSD: p = 0.025). None of the other treatment combinations differed significantly from each other (TukeyHSD: p = 0.078–0.966).

## Discussion

There is intense interest in how the microbiome interacts with host health and physical performance. Probiotics are an appealing therapy for health issues because they are convenient and could alter dysbiotic states to a more favorable one. Probiotics are readily used in humans [51], but studies showing their effectiveness in animals are mixed [27,52]. Here, we conducted a longitudinal study investigating whether pigeon microbiomes are altered by two different popular pigeon probiotics, Blue Seal probiotic pellets and Probios probiotic powder. The control group’s microbiomes did not significantly change over the course of the experiment. Adding probiotic pellets to the diet significantly changed the fecal microbiome of Birmingham Roller pigeons, but we detected no effect of the probiotic powder on the fecal microbiome. We hypothesize that the ineffectiveness of the probiotic powder to shape the microbiome may be due to the administration method: the powder was dissolved in the water of the pigeons, and may not have been ingested in sufficient amounts to cause a detectable change in microbiome.

*Lactobacillus* spp. were among the main ingredients in the probiotic pellets and powder (Table 1). Microbiomes of pigeons that were fed the pellet probiotic increased in lactobacilli abundance, but no change was detected in the other probiotic genus, *Enterococcus*. *E*. *faecium* is often used in mixed probiotic supplements in humans and livestock with positive effects [53,54], but the mechanisms underlying the benefits to its hosts are not as well known. It is possible that the plant-based diet of pigeons could provide better substrates for *Lactobacillus* spp. than for *Enterococcus* spp., and thus giving lactobacilli a competitive advantage. Notably, supplementation of a probiotic containing *Clostridium butyricum*, *Bacillus subtilis*, and *Lactobacillus plantarum* to broiler chickens significantly increased the abundance of lactobacilli, but not the other bacteria, in the cecal microbiome [55]. In a different study, free-living chickens that were fed different probiotics, including enterococci, found that only chickens in the *Lactobacillus* spp. treatment showed a shift in microbiome composition [25]. Thus, *Lactobacillus* spp. may be important members of the avian microbiome that are susceptible to probiotic manipulation and a potential target for therapeutic interventions.

During and immediately following treatment, we detected a significant decrease in two genera that are known to include several pathogens: *Peptostreptococcus* and *Corynebacterium*. *Lactobacillus* spp. in birds are associated with reducing pathogen loads through competitive exclusion and fermentation of different food components such as lactate and plant products [56–60], as well as benefiting health through improving body condition [61]. Pigeon health could therefore be positively affected by probiotic use if the lactobacilli were responsible for the decline in pathogen abundance.

The main Phyla detected in the microbiome of the control pigeons were Actinobacteria (51%), Firmicutes (28%) and Proteobacteria (18%), which all have been documented as major Phyla in other avian taxa [14]. However, such a high abundance of Actinobacteria has only been documented in wild black-legged kittiwakes (*Rissa tridactyla*), a seabird from the Laridae family [62]. Although not as high as in our study, wild ruddy and common ground-doves (*Columbina talpacoti* and *C*. *passerina*) were found to have 21–28% of the microbiome consist of Actinobacteria, which is higher than most avian species including domestic chickens [14,59]. The close phylogenetic relationship to the Birmingham Roller pigeons of these dove species in combination with their similar, granivorous diet could explain our finding of an Actinobacteria-rich microbiome in pigeons.

The pigeon microbiome reverted back to its pre-treatment, Actinobacteria-dominated composition within a day of ceasing the probiotic pellet treatments. Short-term effects of probiotics on microbiome composition have been documented previously but vary considerably depending on hosts and bacterial strains [63–65]. The rapid clearing of the probiotics from the pigeon fecal microbiome we observed raises the question: why is there little to no establishment of *Lactobacillus* spp.? First, it is possible that the supplemented *Lactobacillus* spp. were not able to permanently establish because they were outcompeted by the present microbiome. The competitive ability of *Lactobacilli* in the avian microbiome has not been studied, but in a culturing study with media simulating human infant guts, lactobacilli were strong competitors with microorganisms already present in the infant gut [66]. Conversely, *Lactobacillus* spp. (such as *L*. *gasseri*) were rapidly outcompeted by a *Salmonella enterica* subspecies in co-culture [67], indicating differential responses of lactobacilli to competition with other gut bacteria.

Second, the shift we observed in the fecal microbiome could be the result of oversaturation of the microbiome with probiotics, which was subsequently reflected in the fecal samples. Ingala et al. (2018) showed that the dietary microbiome was disproportionately represented in the fecal microbiome of bats compared to the gut microbiome, which indicates that a shift in fecal microbiome may not necessarily represent a shift in the actual gut [68]. Third, it is possible that colonization did occur, but we did not detect establishment after ceasing probiotic treatments because we examined fecal samples and not gut lining. Although feces more closely reflect the microbiome of the large intestine than other non-lethal sampling methods [69,70], fecal samples were shown to be markedly different from gut mucosal lining in bats [68]. To correct for this potential sample type bias, future studies could sample microbiomes across the length of the GI tract to monitor specific responses to probiotic treatment.

Given how important microbes can be for host biology, it is appealing to identify microbial solutions to health problems and as a way to increase performance in domesticated and wild animals. Here, we have shown that probiotic treatments can affect the microbiome of show pigeons, but the effect may be based on dosage and is not permanent. An obvious next step in this research is to understand how different probiotics—and any associated microbiome shifts—affect the physical performance and health of pigeons.

## Supporting information

S1 Fig Relative abundance of bacterial phyla for two pigeons per treatment groups showing intra-individual variation in the gut microbiome over time. Results are representative of each pigeon treatment (eight total shown for clarity). The horizontal axis represents time, with each bar representing a sampling day with (green) or without (red) probiotic treatment.
(EPS)

S2 Fig Number of observed Amplicon Sequence Variants (ASV’s) in fecal samples collected from domesticated pigeons exposed to different diets. Shaded area represents treatment period, and treatments consisted of Grain (G), Grain + Probiotic Powder (GPow), Grain + Probiotic Pellet (GPel), and Grain + Probiotic Pellet + Probiotic Powder (GPP). The upper left figure shows all treatments; the other three show given treatment vs. the grain-only control for clarity. Error bars represent standard errors, and red stars represent significance at α = 0.05.
(EPS)

S3 Fig **a) Non-multidimensional Scaling (NMDS) of Bray-Curtis distances from fecal microbiomes of domesticated Birmingham Roller pigeons.** Samples collected before treatment (Day 0; blue triangle), at the end of the treatment period (Day 14; black square), and two weeks post-treatment (Day 28; red circle). Powder and Pellet refer to two different probiotic supplements the pigeons received during the treatment period. **b) Non-multidimensional Scaling (NMDS) of unweighted UniFrac distances from fecal microbiomes of domesticated Birmingham Roller pigeons.** Samples collected before treatment (Day 0; blue triangle), at the end of the treatment period (Day 14; black square), and two weeks post-treatment (Day 28; red circle). Powder and Pellet refer to two different probiotic supplements the pigeons received during the treatment period.
(EPS)

S1 Table ASV’s identified as contaminants by the decontam package in R. For ASV sequences, see supplemental excel file decontam.
(DOCX)

S2 Table Sample sizes per treatment per time point (before rarefaction/after rarefaction). (DOCX)

S3 Table Permanova results for three different distances from fecal samples of pigeons on Day 0, 14, and 28 of sampling. Day 0 represents samples collected prior to probiotic treatment, Day 14 during, and Day 28 shows two weeks post treatment.
(DOCX)
