# Correction: Longitudinal microbiome profiling reveals impermanence of probiotic bacteria in domestic pigeons

**DOI:** 10.1371/journal.pone.0220347

**Published:** 2019-07-23

**Authors:** Kirsten Grond, Julie M. Perreau, Wesley T. Loo, A. James Spring, Colleen M. Cavanaugh, Sarah M. Hird

The last three authors, A. James Spring, Colleen M. Cavanaugh, and Sarah M. Hird, should not have been attributed equal contribution to this work. Instead, Colleen M. Cavanaugh (cavanaug@fas.harvard.edu) and Sarah M. Hird (sarah.hird@uconn.edu) should be listed as corresponding authors.
