# Supplementary material for: Longitudinal microbiome profiling reveals impermanence of probiotic bacteria in domestic pigeons
Source: PLoS One. 2019 Jun 17;14(6):e0217804. doi: 10.1371/journal.pone.0217804 (PMC6578490; doi:10.1371/journal.pone.0217804)
Supplement: S1 Table — For ASV sequences, see supplemental excel file decontam. (DOCX) [file pone.0217804.s004.docx]

Table S1. ASV’s identified as contaminants by the decontam package in R. For ASV sequences, see supplemental excel file decontam.xlsx.

| **Phylum** | **Class** | **Order** | **Family** | **Genus** | Species |
| --- | --- | --- | --- | --- | --- |
| Proteobacteria | Betaproteobacteria | Burkholderiales | Comamonadaceae | Delftia | NA |
| Firmicutes | Bacilli | Lactobacillales | Streptococcaceae | Streptococcus | NA |
| Firmicutes | Bacilli | Bacillales | Staphylococcaceae | Staphylococcus | NA |
| Firmicutes | Bacilli | Lactobacillales | Lactobacillaceae | Lactobacillus | NA |
| Proteobacteria | Gammaproteobacteria | Enterobacteriales | Enterobacteriaceae | Pantoea | NA |
| Firmicutes | Bacilli | Bacillales | Planococcaceae | Lysinibacillus | NA |
| Firmicutes | Bacilli | Bacillales | Listeriaceae | Listeria | NA |
| Proteobacteria | Gammaproteobacteria | Pseudomonadales | Moraxellaceae | Acinetobacter | NA |
| Proteobacteria | Gammaproteobacteria | Xanthomonadales | Xanthomonadaceae | Stenotrophomonas | NA |
| Firmicutes | Bacilli | Lactobacillales | Streptococcaceae | Streptococcus | mutans |
| Proteobacteria | Epsilonproteobacteria | Campylobacterales | Helicobacteraceae | Helicobacter | pylori |
| Firmicutes | Clostridia | Clostridiales | Clostridiaceae_1 | Clostridium_sensu_stricto_1 | NA |
| Proteobacteria | Betaproteobacteria | Neisseriales | Neisseriaceae | Neisseria | meningitidis |
| Firmicutes | Clostridia | Clostridiales | Clostridiaceae_1 | Clostridium_sensu_stricto_1 | NA |
| Firmicutes | Bacilli | Lactobacillales | Enterococcaceae | Enterococcus | NA |
| Firmicutes | Bacilli | Lactobacillales | Streptococcaceae | Streptococcus | NA |
| Proteobacteria | Gammaproteobacteria | Pseudomonadales | Moraxellaceae | Moraxella | NA |
| Bacteroidetes | Bacteroidia | Bacteroidales | Bacteroidaceae | Bacteroides | vulgatus |
| Proteobacteria | Gammaproteobacteria | Pseudomonadales | Pseudomonadaceae | Pseudomonas | NA |
| Actinobacteria | Actinobacteria | Corynebacteriales | Corynebacteriaceae | Corynebacterium_1 | NA |
| Firmicutes | Bacilli | Lactobacillales | Streptococcaceae | Streptococcus | NA |
| Actinobacteria | Actinobacteria | Actinomycetales | Actinomycetaceae | Actinomyces | odontolyticus |
| Proteobacteria | Alphaproteobacteria | Rhizobiales | Rhizobiaceae | Rhizobium | NA |
| Deinococcus-Thermus | Deinococci | Deinococcales | Deinococcaceae | Deinococcus | radiodurans |
| Proteobacteria | Gammaproteobacteria | Pseudomonadales | Moraxellaceae | Acinetobacter | NA |
| Proteobacteria | Alphaproteobacteria | Rhodobacterales | Rhodobacteraceae | Rhodobacter | NA |
| Proteobacteria | Alphaproteobacteria | Caulobacterales | Caulobacteraceae | Brevundimonas | NA |
| Firmicutes | Negativicutes | Selenomonadales | Veillonellaceae | Veillonella | NA |
| Proteobacteria | Gammaproteobacteria | Enterobacteriales | Enterobacteriaceae | Klebsiella | NA |
| Proteobacteria | Gammaproteobacteria | Enterobacteriales | Enterobacteriaceae | Morganella | morganii |
| Bacteroidetes | Bacteroidia | Bacteroidales | Bacteroidaceae | Bacteroides | vulgatus |
| Proteobacteria | Gammaproteobacteria | Enterobacteriales | Enterobacteriaceae | Yersinia | NA |
| Actinobacteria | Actinobacteria | Micrococcales | Micrococcaceae | Rothia | dentocariosa |
| Proteobacteria | Betaproteobacteria | Burkholderiales | Burkholderiaceae | Burkholderia-Paraburkholderia | NA |
| Bacteroidetes | Bacteroidia | Bacteroidales | Bacteroidaceae | Bacteroides | vulgatus |
| Firmicutes | Bacilli | Bacillales | Bacillaceae | Bacillus | NA |
| Proteobacteria | Alphaproteobacteria | Rhodospirillales | Rhodospirillaceae | Azospirillum | NA |
| Bacteroidetes | Bacteroidia | Bacteroidales | Porphyromonadaceae | Porphyromonas | gingivalis |
| Proteobacteria | Gammaproteobacteria | Enterobacteriales | Enterobacteriaceae | NA | NA |
| Firmicutes | Clostridia | Clostridiales | Clostridiaceae_1 | Clostridium_sensu_stricto_1 | beijerinckii |
| Proteobacteria | Gammaproteobacteria | Pseudomonadales | Pseudomonadaceae | NA | NA |
| Firmicutes | Negativicutes | Selenomonadales | Veillonellaceae | Veillonella | NA |
| Proteobacteria | Betaproteobacteria | Burkholderiales | Burkholderiaceae | Lautropia | mirabilis |
| Firmicutes | Bacilli | Bacillales | Bacillaceae | Bacillus | NA |
| Actinobacteria | Actinobacteria | Propionibacteriales | Propionibacteriaceae | Propionibacterium | NA |
| Firmicutes | Bacilli | Bacillales | Family_XI | Gemella | NA |
| Proteobacteria | Alphaproteobacteria | Sphingomonadales | Sphingomonadaceae | Sphingomonas | dokdonensis |
| Proteobacteria | Betaproteobacteria | Burkholderiales | Comamonadaceae | NA | NA |
| Actinobacteria | Actinobacteria | Micrococcales | Micrococcaceae | Kocuria | NA |
| Actinobacteria | Actinobacteria | Bifidobacteriales | Bifidobacteriaceae | Bifidobacterium | NA |
| Firmicutes | Clostridia | Clostridiales | Family_XI | Peptoniphilus | NA |
| Proteobacteria | Alphaproteobacteria | Sphingomonadales | Sphingomonadaceae | Sphingobium | yanoikuyae |
| Bacteroidetes | Flavobacteriia | Flavobacteriales | Flavobacteriaceae | Chryseobacterium | NA |
| Firmicutes | Bacilli | Bacillales | Staphylococcaceae | Staphylococcus | NA |
| Deinococcus-Thermus | Deinococci | Thermales | Thermaceae | Thermus | amyloliquefaciens |
| Proteobacteria | Betaproteobacteria | Burkholderiales | Oxalobacteraceae | Massilia | NA |
| Bacteroidetes | Sphingobacteriia | Sphingobacteriales | Chitinophagaceae | NA | NA |
| Proteobacteria | Alphaproteobacteria | Rhizobiales | Methylobacteriaceae | Methylobacterium | radiotolerans |
| Firmicutes | Bacilli | Lactobacillales | Carnobacteriaceae | Granulicatella | NA |
| Proteobacteria | Betaproteobacteria | Burkholderiales | Burkholderiaceae | Ralstonia | NA |
| Bacteroidetes | Bacteroidia | Bacteroidales | Prevotellaceae | Prevotella_7 | veroralis |
| Proteobacteria | Alphaproteobacteria | Rhizobiales | Methylobacteriaceae | Methylobacterium | aerolatum |
| Bacteroidetes | Bacteroidia | Bacteroidales | Prevotellaceae | Prevotella | nigrescens |
| Bacteroidetes | Cytophagia | Cytophagales | Cytophagaceae | Emticicia | NA |
| Actinobacteria | Actinobacteria | Actinomycetales | Actinomycetaceae | Actinomyces | massiliensis |
| Firmicutes | Bacilli | Lactobacillales | Lactobacillaceae | Lactobacillus | iners |
| Firmicutes | Bacilli | Lactobacillales | Streptococcaceae | Streptococcus | NA |
| Actinobacteria | Actinobacteria | Actinomycetales | Actinomycetaceae | Actinomyces | NA |
| Proteobacteria | Gammaproteobacteria | Xanthomonadales | Xanthomonadales_Incertae_Sedis | Acidibacter | NA |
| Proteobacteria | Alphaproteobacteria | Rickettsiales | Mitochondria | NA | NA |
| Proteobacteria | Betaproteobacteria | Burkholderiales | Oxalobacteraceae | Duganella | NA |
| Fusobacteria | Fusobacteriia | Fusobacteriales | Leptotrichiaceae | Leptotrichia | NA |
| Proteobacteria | Betaproteobacteria | Burkholderiales | Alcaligenaceae | Achromobacter | NA |
| Firmicutes | Clostridia | Clostridiales | Lachnospiraceae | NA | NA |
| Bacteroidetes | Bacteroidia | Bacteroidales | Porphyromonadaceae | Proteiniphilum | acetatigenes |
| Proteobacteria | Alphaproteobacteria | Rhizobiales | Bradyrhizobiaceae | Rhodopseudomonas | boonkerdii |
| Firmicutes | Negativicutes | Selenomonadales | Veillonellaceae | Megasphaera | micronuciformis |
| Proteobacteria | Alphaproteobacteria | Rhodobacterales | Rhodobacteraceae | Rubellimicrobium | roseum |
| Bacteroidetes | Bacteroidia | Bacteroidales | Prevotellaceae | Prevotella | buccalis |
| Actinobacteria | Actinobacteria | Actinomycetales | Actinomycetaceae | Actinomyces | odontolyticus |
| Proteobacteria | Alphaproteobacteria | Rickettsiales | Mitochondria | NA | NA |
| Proteobacteria | Alphaproteobacteria | Rhizobiales | Aurantimonadaceae | Martelella | NA |
| Bacteroidetes | Bacteroidia | Bacteroidales | Prevotellaceae | Prevotella | pallens |
| Bacteroidetes | Bacteroidia | Bacteroidales | Prevotellaceae | Alloprevotella | NA |
| Actinobacteria | Thermoleophilia | Solirubrobacterales | Patulibacteraceae | Patulibacter | americanus |
| Proteobacteria | Gammaproteobacteria | Xanthomonadales | Xanthomonadaceae | Luteibacter | rhizovicinus |
| Bacteroidetes | Flavobacteriia | Flavobacteriales | Flavobacteriaceae | Capnocytophaga | granulosa |
