# Supplementary material for: Longitudinal microbiome profiling reveals impermanence of probiotic bacteria in domestic pigeons
Source: PLoS One. 2019 Jun 17;14(6):e0217804. doi: 10.1371/journal.pone.0217804 (PMC6578490; doi:10.1371/journal.pone.0217804)
Supplement: S2 Table — (DOCX) [file pone.0217804.s005.docx]

Table S2. Sample sizes per treatment per time point (before rarefaction/after rarefaction).

| Sampling Day | Grain (G) | Grain + Powder (Gpo) | Grain + Pellet (Gpe) | Grain + Powder + Pellet (GPP) |
| --- | --- | --- | --- | --- |
| 0 | 6 / 5 | 7 / 6 | 7 / 5 | 7 / 7 |
| 1 | 3 / 2 | 5 / 5 | 5 / 5 | 5 / 5 |
| 3 | 4 / 3 | 5 / 4 | 5 / 4 | 5 / 5 |
| 5 | 5 / 5 | 5 / 4 | 5 / 5 | 5 / 5 |
| 9 | 3 / 3 | 5 / 3 | 5 / 5 | 5 / 5 |
| 14 | 5 / 5 | 5 / 5 | 5 / 4 | 5 / 5 |
| 15 | 5 / 5 | 4 / 3 | 5 / 3 | 4 / 4 |
| 17 | 4 / 3 | 5 / 4 | 5 / 4 | 5 / 4 |
| 19 | 5 / 5 | 4 / 4 | 3 / 3 | 5 / 5 |
| 23 | 5 / 5 | 3 / 3 | 5 / 5 | 5 / 4 |
| 28 | 5 / 4 | 5 / 5 | 5 / 3 | 5 / 5 |
| 42 | 4 / 4 | 5 / 4 | 4 / 3 | 5 / 5 |
