# Supplementary material for: Longitudinal microbiome profiling reveals impermanence of probiotic bacteria in domestic pigeons
Source: PLoS One. 2019 Jun 17;14(6):e0217804. doi: 10.1371/journal.pone.0217804 (PMC6578490; doi:10.1371/journal.pone.0217804)
Supplement: S3 Table — Day 0 represents samples collected prior to probiotic treatment, Day 14 during, and Day 28 shows two weeks post treatment. (DOCX) [file pone.0217804.s006.docx]

Table S3. Permanova results for three different distances from fecal samples of pigeons on Day 0, 14, and 28 of sampling. Day 0 represents samples collected prior to probiotic treatment, Day 14 during, and Day 28 shows two weeks post treatment.

|  | Bray-Curtis | | Unweighted UniFrac | | Weighted UniFrac | |
| --- | --- | --- | --- | --- | --- | --- |
|  | R^2^ | p | R^2^ | p | R^2^ | p |
| Day 0 | 0.094 | 0.74 | 0.064 | 0.99 | 0.140 | 0.282 |
| Day 14 | 0.364 | **< 0.001** | 0.264 | **0.011** | 0.465 | **0.002** |
| Day 28 | 0.164 | 0.369 | 0.238 | 0.069 | 0.145 | 0.477 |
